# Trust yourself, discover your resilience! From self-efficacy to life satisfaction: the mediating effect of psychological resilience

**DOI:** 10.3389/fpsyg.2025.1697876

**Published:** 2025-11-19

**Authors:** Burak Karababa, Gokcer Aydin, Oguzhan Yilmaz, Yavuz Akkus, Esra Bayram, Gokhan Aydin

**Affiliations:** 1 Department of Physical Education and Sports, Sport Sciences Faculty, Erzurum Technical University, Erzurum, Türkiye; 2 Department of Sports Management, Graduate School of Winter Sports and Sport Sciences, Ataturk University, Erzurum, Türkiye; 3 Department of Physical Education and Sports, Sport Sciences Faculty, Ataturk University, Erzurum, Türkiye; 4 Department of Physical Education and Sports, Graduate School of Winter Sports and Sport Sciences, Ataturk University, Erzurum, Türkiye; 5 Department of Sports Management, Sport Sciences Faculty, Ataturk University, Erzurum, Türkiye

**Keywords:** self-efficacy, psychological resilience, life satisfaction, sport, student athletes

## Abstract

**Introduction:**

This study investigates the mediating role of psychological resilience in the relationship between self-efficacy and life satisfaction among student athletes. Self-efficacy and psychological resilience are considered key psychological resources that can enhance overall well-being and life satisfaction, particularly in the context of sports.

**Methods:**

The research was conducted using a quantitative design. The sample consisted of 481 student athletes (223 male, 46.4%; 258 female, 53.6%) studying at faculties of sport sciences in various universities across Turkey. Data were collected through the General Self-Efficacy Scale, the Brief Resilience Scale, and the Life Satisfaction Scale. Statistical analyses were performed using SPSS 27.0 and the PROCESS Macro (Model 4). Pearson correlation analysis was used to examine relationships between variables, followed by mediation analyses based on hierarchical regression and bootstrap methods.

**Results:**

Findings indicated that self-efficacy significantly and positively predicted psychological resilience (*β* = 0.517, *p* < 0.001). Both self-efficacy (*β* = 0.278, *p* < 0.01) and psychological resilience (*β* = 0.141, *p* < 0.01) were significant predictors of life satisfaction. Mediation analysis revealed that psychological resilience partially mediated the relationship between self-efficacy and life satisfaction (*β* = 0.073, 95% CI [0.024, 0.126]). The measurement model demonstrated acceptable fit indices (*χ*²/df = 2.661; RMSEA = 0.059; CFI = 0.943; TLI = 0.932).

**Discussion:**

These results highlight the importance of self-efficacy and psychological resilience as fundamental factors that promote life satisfaction among university student athletes. The study suggests that sports environments can enhance life satisfaction by strengthening both physical and psychological resources. Based on these findings, developing intervention programs aimed at improving self-efficacy and psychological resilience may significantly contribute to the well-being and satisfaction of student athletes.

## Introduction

University-aged athletes are exposed to various stress factors and depressive symptoms due to their academic and athletic commitments, which may influence key psychological variables such as life satisfaction (Beisecker et al., 2024; Tolukan et al., 2024). Compared to the general student population, university athletes experience greater psychological pressure as they must simultaneously meet academic and athletic expectations. Research in Turkey shows that depressive symptoms are widespread among university students, generally affecting 20–30% of them (Bayram and Bilgel, 2008; Kaya et al., 2007). Recent studies, however, indicate that these rates have risen to 35–40% in the post-pandemic period (Aslan and Çınar, 2023; Cam et al., 2022). Moreover, anxiety, burnout, and depression symptoms tend to be more common among student-athletes than in the general population (Weber et al., 2023; Xu et al., 2025). Economic difficulties and limited employment prospects have further intensified this problem, with approximately one in four students experiencing depressive symptoms (Wang and Liu, 2022). Such stressors reduce life satisfaction and increase psychological distress (Shan et al., 2022). Despite these findings, quantitative data specifically focusing on stress, depression, and resilience among university student-athletes remain scarce. Expanding up-to-date research in this area is essential for understanding their psychological profiles and developing effective interventions.

A similar pattern appears in the few studies conducted with Turkish student-athletes. For instance, Tolukan et al. (2024) found clinical-level stress in about one-third of participants. Other studies have shown that stress and burnout rise when athletes face conflicting academic and athletic demands (Lopes Dos Santos et al., 2020; Xu et al., 2025). These results highlight the need for research focusing on this specialized population within its sociocultural and educational context, offering original insights to the literature.

The restorative role of sports is particularly important in this context. Previous research shows that participation in sports reduces depressive symptoms and stress while enhancing life satisfaction, self-efficacy, and psychological resilience (Lubans et al., 2016; McDowell et al., 2019; Zhang et al., 2020). Resilience is a physical quality of “endurance” and the ability to adapt and recover psychologically in the face of difficulties. It reflects how individuals use emotional, cognitive, and behavioral resources to maintain functionality. Regular physical activity has been shown to strengthen self-efficacy and resilience, improving university athletes’ life satisfaction (Deng et al., 2023; Zhang, 2025). Thus, self-efficacy and psychological resilience are key mechanisms that help athletes cope with academic and athletic stressors while sustaining life satisfaction.

The theoretical foundation of this study is based on Bandura’s Social Cognitive Theory (1997) and Self-Determination Theory (Deci and Ryan, 2000). Social Cognitive Theory emphasizes the importance of self-efficacy in managing environmental challenges. At the same time, Self-Determination Theory underlines the fulfillment of autonomy, competence, and relatedness as central to motivation and wellbeing. Integrating these theories provides a comprehensive view of how motivational and self-regulatory processes interact to shape psychological outcomes. Considering the Turkish context—where collectivist norms, family expectations, and social obligations significantly influence motivation—this framework gains additional cultural relevance. According to this model, individuals with high self-efficacy evaluate stressors constructively, enhancing psychological resilience, consistent with the Cognitive Appraisal Model (Lazarus and Folkman, 1984). Resilience thus serves as a mediating mechanism that transforms self-efficacy into life satisfaction. By incorporating cognitive appraisal and self-regulation perspectives, the present study proposes a multidimensional and culturally grounded model for understanding student-athlete life satisfaction.

Life satisfaction is a subjective measure of overall wellbeing and is closely associated with psychological health, academic achievement, social relationships, and physical wellness (Pavot and Diener, 2008). High life satisfaction helps individuals manage stress, maintain motivation, and consistently pursue long-term goals (Lent, 2013). For student-athletes, life satisfaction is a crucial indicator of psychological wellbeing, affecting performance and persistence in sports (Gillet et al., 2012; Jowett and Cramer, 2010). Within Self-Determination Theory, life satisfaction reflects how individuals’ basic psychological needs are fulfilled, a process supported by internal resources like self-efficacy and resilience.

In Turkey, the psychological experiences of university athletes are shaped by multiple cultural, social, and economic factors. The pressures they face extend beyond academic and athletic demands. Competition, limited scholarships, uncertainties about professional sports careers, and family expectations contribute to stress (Bilgin and Taş, 2018). Post-pandemic challenges such as financial insecurity, reduced motivation, and social isolation have also weakened athletes’ psychological resilience (Akgül et al., 2023; Bölükbaş et al., 2022). These findings show how cultural and structural dynamics shape Turkish student-athletes’ emotional responses and coping behaviors.

The prioritization of academic success over athletic achievement often creates identity conflicts for student-athletes, heightening their need for personal resources like self-efficacy and resilience. Contextual factors such as social support and identity influence their self-regulation and coping strategies (Carver and Scheier, 2001). Social support is an external protective resource, while self-regulatory processes sustain goal-directed behavior. In collectivist societies like Turkey, family cohesion and peer support play a central role in fostering resilience and reducing stress. Understanding these cultural dynamics provides valuable cross-cultural insights into sport psychology.

Turkey’s collectivist culture shapes how individuals use social support, making family and peer relationships key protective factors in coping with stress (Öztürk and Kundakçı, 2021). To explain life satisfaction among university athletes, it is necessary to consider these cultural dimensions alongside the role of sport in strengthening resilience. Focusing on these culturally embedded mechanisms, this study seeks to enhance theoretical and empirical understanding of resilience in a non-Western athletic context. Accordingly, the present study provides a unique contribution by situating its analysis within Turkey’s social and cultural environment.

As defined by Bandura (1997), self-efficacy refers to the belief in one’s ability to complete a specific task. Individuals with high self-efficacy view challenges as opportunities rather than obstacles, and this belief influences their motivation, emotions, and goal-directed behaviors. Studies have found that self-efficacy is positively associated with self-esteem, positive emotions, and motivation (Dumangöz and Sanlav, 2021). Athletes with strong self-efficacy tend to perform better and maintain higher psychological wellbeing by approaching difficulties constructively (Aizava et al., 2023). Self-efficacy has also been shown to predict life satisfaction, explaining nearly half of its variance in young adults (Çakar, 2012). Recent findings confirm that self-efficacy significantly determines motivation, self-regulation, and wellbeing in student-athletes (Aizava et al., 2023; Biscardi et al., 2024; Qin et al., 2023).

Psychological resilience refers to the capacity to adapt and recover from stress and adversity, emphasizing flexibility rather than mere resistance. High resilience contributes significantly to life satisfaction (Kiylioglu, 2024). In Turkey, family cohesion, community support, and cultural solidarity are central elements shaping young people’s resilience (Türk et al., 2025). These protective factors illustrate how individual and collective strengths interact in the development of resilience. Studies with athlete samples also show that resilience mediates the relationship between psychological wellbeing and performance (Ancarani et al., 2024; Yang et al., 2023; Zhang and Li, 2025). Thus, resilience represents the effective use of internal (self-efficacy, self-regulation) and external (social support, cultural solidarity) resources.

Previous research indicates a positive relationship between self-efficacy and resilience, directly and indirectly influencing life satisfaction (Arslan and Balkıs, 2016; Schueler et al., 2021; Yu et al., 2025). While self-efficacy acts as a personal resource enhancing life satisfaction, resilience is a mediating factor in this process (Deng et al., 2023; Durak, 2021; Qin et al., 2023). The present study extends existing research by examining this mediation model among Turkish university athletes, offering new empirical insights into an underexplored group. Accordingly, this study contributes to the literature by testing the mediating effect of resilience on the relationship between self-efficacy and life satisfaction within a specific cultural context.

In this context, the following hypotheses were developed for the study:

*H1*: Self-efficacy levels positively predict life satisfaction.

*H2*: Self-efficacy levels positively predict psychological resilience.

*H3*: Psychological resilience levels positively predict life satisfaction.

*H4*: Psychological resilience plays a mediating role in the relationship between self-efficacy and life satisfaction.

**Figure 1:**
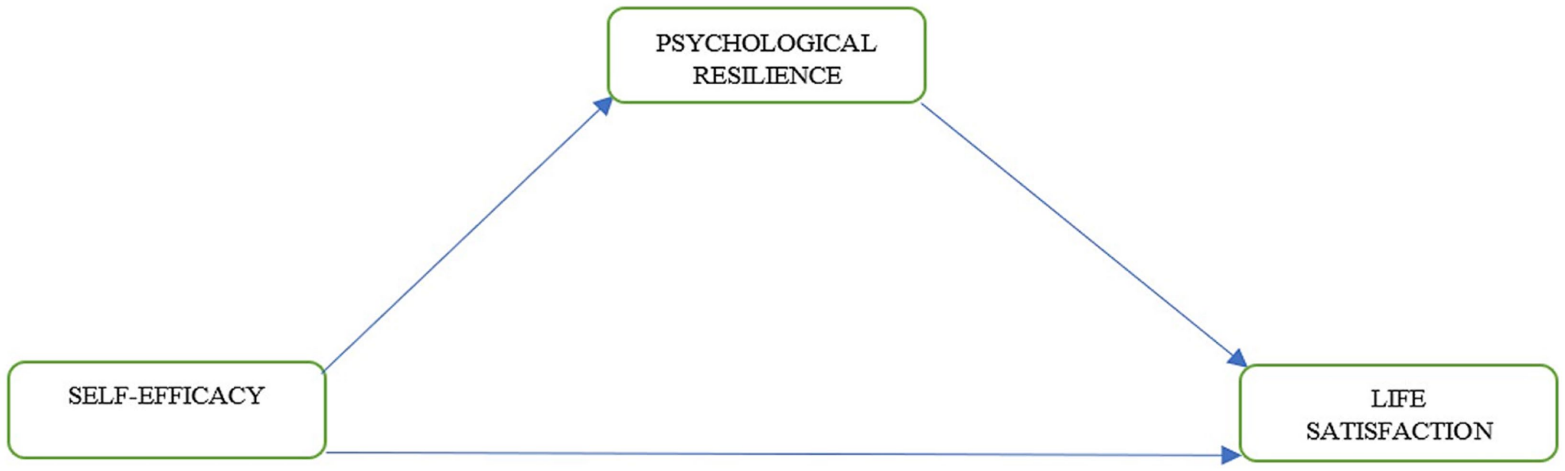
Proposed research model.

The above hypotheses constitute the conceptual model of the study. Therefore, the proposed research model is summarized in Figure 1, which visually depicts the study’s conceptual framework and the relationships among the hypotheses.

**Figure 2:**
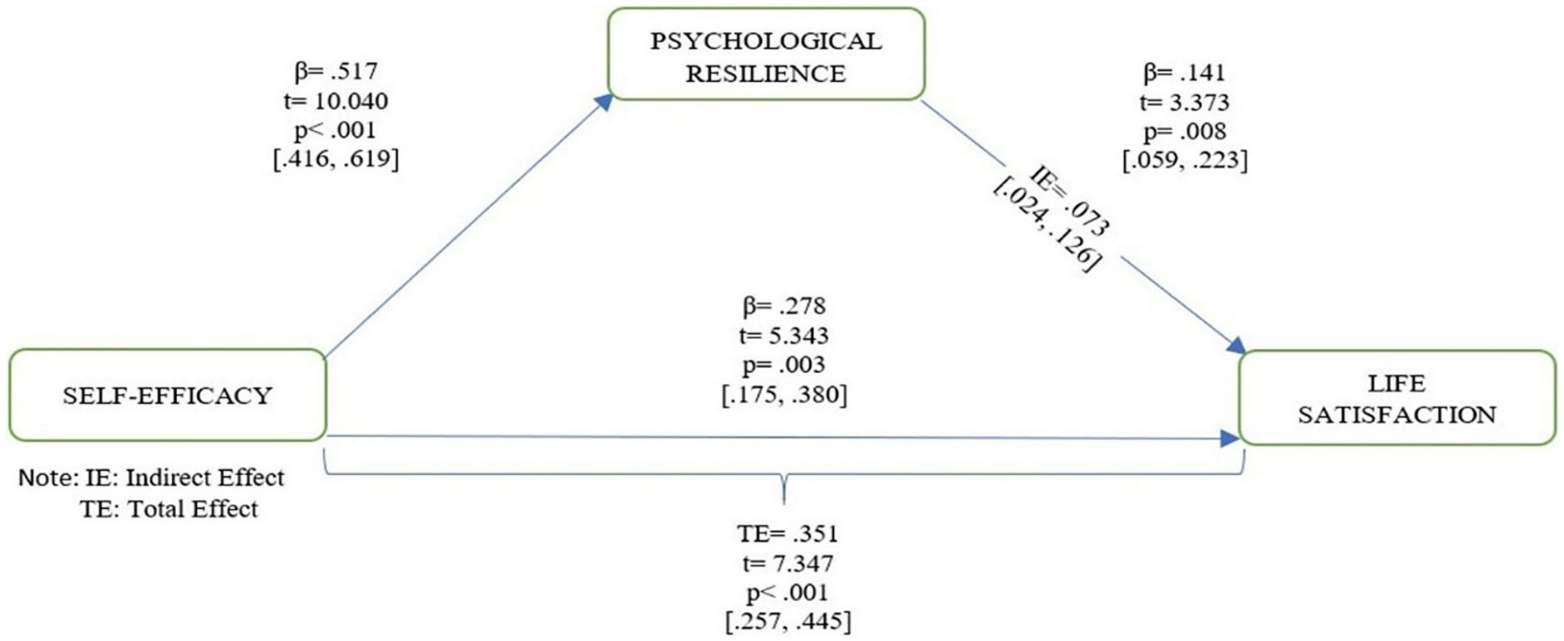
Model of the mediating role of psychological resilience in the relationship between self-efficacy and life satisfaction.

## Methods

### Participants

A power analysis was performed using G*Power 3.1 to determine the required sample size. The analysis, based on multiple regression (f^2^ = 0.05, medium effect size), was conducted with a significance level of 0.05 and a statistical power of 0.80. The medium effect size was determined according to Cohen’s (2013) standards and supported by previous studies (Aypay, 2010; Doğan, 2015; Lam, 2012). Results indicated that at least 162 participants were needed to test Path a (MX) and Path b (YX + M) in the mediation model. For higher power (0.90), approximately 216 participants were required. Accordingly, 481 student-athletes from faculties of sport sciences in Turkey participated in the study. Participants were voluntarily selected from randomly chosen class groups based on faculty student lists, representing a volunteer-based subtype of probability sampling. In this procedure, class groups were randomly selected to ensure diversity, and within these groups, participation was entirely voluntary. Therefore, the sampling approach combined random selection at the group level with voluntary participation at the individual level, aiming to enhance representativeness while respecting ethical principles of voluntariness. Data were collected using paper-based questionnaires. Among the participants, 223 (46.4%) were male and 258 (53.6%) were female. By grade level, 180 (37.4%) were first-year, 139 (28.9%) second-year, 94 (19.5%) third-year, and 68 (14.2%) fourth-year students. The mean age was 21.7 years (SD = 3.2), and the mean duration of sports participation was 4.8 years (SD = 4.1).

### Data collection tools

#### Life satisfaction scale

The Life Satisfaction Scale was used to determine individuals’ levels of life satisfaction. The scale was developed by Lavallee et al. (2007) and adapted to Turkish by Akın and Yalnız (2015). Items 3 and 4 in this single-dimensional scale, with 5 items, were reverse-coded. The scale is a 7-point Likert-type scale consisting of items “strongly disagree (1) and strongly agree (7).” Cronbach’s alpha reliability coefficient was calculated as 0.87 in the original form of the scale. On the other hand, Akın and Yalnız (2015) calculated the scale’s Cronbach’s alpha coefficient as 0.73 in their study. In this study, the Cronbach’s alpha coefficient was calculated as 0.70.

#### Brief resilience scale

Smith et al. (2008) developed the Brief Resilience Scale to measure individuals’ capacity to recover psychologically from stressful life events. The scale does not directly measure constructs such as ego resilience, optimism, or social support; however, it can be linked to these concepts within the theoretical framework related to psychological resilience. The Brief Resilience Scale is a measurement tool comprising 6 items scored using a 5-point Likert scale (1- Strongly Disagree; 5- Strongly Agree). Items 2, 4, and 6 were reverse-coded, and this reverse coding was confirmed in the Turkish adaptation study (Doğan, 2015). Higher scores obtained from the scale indicate higher psychological resilience. The Turkish validity and reliability study of the scale was conducted by Doğan (2015), who calculated the Cronbach’s alpha coefficient as 0.83. In this study, Cronbach’s alpha coefficient was calculated as 0.71. This relatively lower alpha value may be due to several factors. First, cultural adaptation and contextual differences can influence how certain items are interpreted within the Turkish university athlete population. Second, the homogeneous nature of the sample (student-athletes from similar academic and sports environments) may have reduced response variability. Finally, as the BRS includes only six items, shorter scales often yield slightly lower reliability coefficients due to item limitation effects. Despite this, the obtained value 0.71 remains within the acceptable threshold, supporting the reliability of the instrument for the current study.

#### General self-efficacy scale

The general self-efficacy scale, developed by Schwarzer and Jerusalem (1995), was adapted into Turkish by Aypay (2010). The scale, consisting of 10 items and a single sub-dimension, was a valid and reliable tool for measuring general self-efficacy. The GÖYÖ is a 4-point Likert-type scale. The degree of agreement with the scale is expressed as (1) “Completely false,” (2) “Somewhat true,” (3) “Moderately true,” (4) “Completely true.” Cronbach’s alpha coefficient for the scale was calculated as 0.83. In this study, Cronbach’s alpha coefficient was calculated as 0.88.

#### Procedure

The research was conducted with the approval of the Ethics Committee of the Faculty of Sport Sciences, Atatürk University (Date: July 17, 2025; Decision No: E-70400699-050.02.04-2500239949/134), ensuring that the study complied with scientific standards and national/international ethical principles. A written “Informed Consent Form” was provided to the participants before the research began. This form clearly stated the purpose of the study, its duration, potential risks and benefits, the principle of voluntariness, the confidentiality of the data, and the use of the data solely for scientific purposes. Data collection was carried out before the final examinations of the 2024–2025 Summer School Term, which minimized potential fluctuations in participants’ life satisfaction and self-efficacy perceptions related to exam stress. Data collection was carried out with the voluntary participation of student athletes studying at the Faculty of Sport Sciences. Surveys were administered in quiet, distraction-free classrooms. The research team physically distributed the surveys, and participants completed the forms simultaneously and under supervision (but with the protection of individual confidentiality). Participants were provided with detailed information about the study’s purpose, scope, and process. It was clearly stated that participation in the study was entirely voluntary, that the data obtained would be used solely for scientific purposes, and that personal information would not be shared with third parties. To maintain the confidentiality of the data, no personal identification information (name, student number, etc.) was collected; all responses were coded anonymously. The data obtained was stored in an encrypted digital environment, accessible only to the researchers, and was not shared with third parties. Before administering the data collection instruments, participants were provided with detailed and clear instructions on completing the survey. The researchers ensured that all participants participated in the data collection process under the same conditions and created a suitable environment for participants to focus their attention throughout the application. Participants completed the survey instruments in approximately 15 min. To mitigate social desirability effects, it was emphasized that answers were not right or wrong; only individual experiences and perceptions mattered. Furthermore, the fact that responses would be evaluated anonymously reinforced motivation for honest responses. This aimed to ensure that the data obtained reflected participants’ accurate perceptions and experiences.

#### Data analysis

This research was conducted using a relational screening design within the framework of a quantitative research approach. The relational screening model is a research design that aims to reveal the existence and direction of relationships between two or more variables (Karasar, 2007). Data analysis used the SPSS 27.0 package program, AMOS Graphics software, and PROCESS Macro (Model 4) (Hayes, 2017). Before proceeding with the analyses, the data set was examined for missing values and outlier observations, and the necessary cleaning procedures were performed. After the mean values of the variables were obtained, z scores were calculated for each and included in the model. Z-scores were calculated to address comparability issues arising from the use of different Likert scale ranges (4-, 5-, and 7-point) and were used in the regression and mediation analyses. In descriptive statistics, raw scores were reported to preserve the interpretability of the original scales. The use of z-score transformation ensured that variables measured on different scale ranges could be meaningfully compared within the same model. This standardization does not change the direction or statistical significance of the relationships but enables all coefficients to be interpreted on a standardized scale. Thus, the transformation served a methodological purpose of comparability rather than altering substantive interpretation. Standardization was not applied to correlation analyses, as correlation coefficients are inherently independent of measurement units. Descriptive statistics showed that life satisfaction (M = 4.28, SD = 0.67, range = 1–7), psychological resilience (M = 3.23, SD = 0.75, range = 1–5), and self-efficacy (M = 3.08, SD = 0.61, range = 1–4) were at relatively high, moderate, and strong levels, respectively. Thus, the beta coefficients obtained are standardized values. This method provided integrity in interpretation by balancing scaling differences that may arise from direct comparison of different scale formats (4-, 5-, and 7-point Likert). The internal consistency reliability of the scales was assessed using Cronbach’s Alpha coefficients and was found to be at an acceptable level.

Although demographic factors such as age, gender, and years of athletic experience are known to influence self-efficacy and life satisfaction, they were not included as control variables in the current analysis. The study aimed to test the general structural relationships among psychological resilience, self-efficacy, and life satisfaction, focusing on psychological mechanisms rather than individual demographic differences. Moreover, because the sample consisted of university student athletes with relatively similar demographic backgrounds, potential variability due to these factors was assumed to be minimal.

The study implemented preventive procedures during the data collection to reduce standard method variance (CMV). Participants were clearly informed that the study was anonymous, that their responses would be kept confidential, and that they would not be used for evaluation or classification purposes in any way. Furthermore, the measurement tools used scale types (4-, 5-, and 7-point Likert-type) to reduce the single-source effect, randomized the order of scale items, and used different response formats for different variables. These practices aimed to reduce systematic response biases and increase the validity of the findings. In addition, Harman’s Single Factor Test was also applied to assess the risk of standard method bias more comprehensively despite these measures. Unrotated factor analysis (Principal Component Analysis) was performed on all scale items, revealing that the first factor explained 31.38% of the total variance. Since this ratio was well below 50%, it was concluded that standard method variance did not seriously threaten the study (Podsakoff et al., 2003). For future studies, employing alternative strategies—such as incorporating behavioral measures, collecting data at different time points, or using multiple data sources—is recommended to further minimize the potential risk of common method variance and enhance methodological rigor.

The normality assumption was assessed through visual inspection of P–P plots along with the skewness and kurtosis values of the variables. The skewness value for self-efficacy was −0.300, and the kurtosis value was −0.386; the skewness value for psychological resilience was 0.154, and the kurtosis value was 0.515; and the skewness value for life satisfaction was 0.019, and the kurtosis value was 0.451. All values were within ±1.5, indicating a near-normal distribution of the data. These findings demonstrate that the distributions did not deviate excessively from normality and confirm that the normality assumption was met (Tabachnick and Fidell, 2019).

The Variance Increment Factor (VIF) and tolerance values were examined to assess multicollinearity among the independent variables. The analysis results revealed that the values for self-efficacy (VIF = 1.210, Tolerance = 0.826) and psychological resilience (VIF = 1.210, Tolerance = 0.826) were within acceptable limits, and the Condition Index (12.533) value was well below the critical threshold of 30 (Hair et al., 2009). These findings indicate that the model did not exhibit multicollinearity.

In order to assess the validity of the proposed model in the study, Confirmatory Factor Analyses (CFA) were conducted using the AMOS Graphics program, and the obtained fit indices were determined to be at an acceptable level. Pearson correlation analysis was conducted to examine the relationships between variables; hierarchical regression analysis and indirect effect analyses based on the bootstrap method were conducted to test the mediating role of psychological resilience in the relationship between self-efficacy and life satisfaction. A total of 5,000 resamples were used in the bootstrap analyses, and the significance of the mediating effect was tested with 95% confidence intervals (LLCI–ULCI). The criterion for accepting an indirect effect as statistically significant was that the confidence interval did not include zero. The overall explanatory power of the model was evaluated using the coefficient of determination (R^2^) and the relevant *F* values. In addition, within the scope of the validity and reliability analyses of the measurement tools used in the study, Composite Reliability (CR), Average Variance Extraction (AVE), and Fornell and Larcker (1981) discriminant validity criteria were examined. The results showed that the scales used were valid and reliable measurement tools. A significance level of *p <* 0.05 was accepted in all analyses (Table 1).

**Table 1:** Reliability, validity, and correlation results of the scales.

Variable	CR	AVE	√AVE	1	2	3	F & L
1. SE	0.889	0.445	0.667	1			✓
2. PR	0.694	0.442	0.665	0.481**	1		✓
3. LS	0.872	0.577	0.760	0.318**	0.264**	1	✓

SE, Self-efficacy; PR, Psychological Resilience; LS, Life Satisfaction; CR, Composite Reliability; AVE, Average Variance Extracted; F & L, Fornell & Larcker Criterion (1981); **p < 0.01.

## Results

The findings obtained from the validity and reliability analyses of the scales used in the study indicate that the scales are broadly acceptable. First, when the composite reliability (CR) values are examined, it is seen that SE (0.889) and LS (0.872) are above the recommended threshold of 0.70 and exhibit high internal consistency. The PS (0.694) variable is close to 0.70 but is acceptable (Hair et al., 2009). Regarding average variance extraction (AVE), the requirement of being above 0.50, as accepted in the literature, was met only for the LS (0.577) variable. In contrast, the AVE values of SE (0.445) and PR (0.442) variables were below 0.50, indicating limitations in terms of convergent validity. Although the AVE values for self-efficacy and psychological resilience fell below the recommended threshold, this limitation may be attributed to several factors. First, some items—particularly in the PR scale—showed relatively lower factor loadings, which may have reduced the overall variance explained by the latent construct. Second, the scales were adapted to a different cultural context, and minor semantic or conceptual shifts could have affected the internal consistency of certain items. Furthermore, psychological resilience is theoretically a multidimensional construct involving emotional, cognitive, and behavioral components, which may not be fully captured by a unidimensional measurement model. These factors may partly explain the lower AVE values observed. Nonetheless, given that the composite reliability and discriminant validity indices are within acceptable limits, the measurement model remains conceptually sound and statistically robust. However, when the discriminant validity criterion suggested by Fornell and Larcker (1981) is examined, it is seen that the √AVE values (SE = 0.667, PR = 0.665, LS = 0.760) of each construct are higher than the correlation values with other constructs. Therefore, the model provides discriminant validity, but convergent validity is limited, particularly in the PR dimension. The relatively low convergent validity (AVE) values for the self-efficacy and psychological resilience variables in the measurement model, compared to the recommended threshold of 0.50, were carefully evaluated for model validity. However, as Hair et al. (2009) and Lam (2012) noted, low AVE values alone do not necessarily indicate a model’s inadequacy. Construct validity is acceptable, especially when composite reliability (CR) values exceed 0.70. In this context, discriminant validity analyses were also conducted to support the model’s overall validity, and the HTMT ratio was calculated in addition to the Fornell–Larcker criterion. The results indicate that all HTMT values are below 0.85, and the constructs are distinguishable. These findings reveal that the measurement model maintains its theoretical consistency, that convergent validity limitations do not undermine the overall structural validity of the model, and that the model is statistically interpretable in its current form (Hair et al., 2009; Henseler et al., 2015; Lam, 2012).

Table 2 presents the Heterotrait–Monotrait (HTMT) ratios among constructs, with the square root of the Average Variance Extracted (√AVE) values shown on the diagonal. All HTMT ratios are below the threshold of 0.85 (Henseler et al., 2015), indicating that discriminant validity is established among the constructs in the model. Moreover, the √AVE values on the diagonal are higher than the inter-construct correlations, confirming that each construct represents a distinct underlying concept (Table 2).

**Table 2:** HTMT values with √AVE on the diagonal.

Constructs	SE	PR	LS
SE	0.667		
PR	0.628	0.665	
LS	0.583	0.541	0.760

SE, Self-efficacy; PR, Psychological Resilience; LS, Life Satisfaction.

The overall goodness-of-fit indices of the model were calculated as *χ*^2^/df = 2.661, RMSEA = 0.059, CFI = 0.943, GFI = 0.929, NFI = 0.912, TLI = 0.932, and IFI = 0.943. In the literature (Hair et al., 2009; Hu and Bentler, 1999; Kline, 2023), it is stated that a *χ*^2^/df value below 3, an RMSEA value below 0.08, and CFI, TLI, IFI, and NFI values above 0.90 indicate a good fit. In this context, all fit indices of the model are above the recommended limits, and it can be said that the overall fit is good. The relatively proximity of the GFI (0.929) and NFI (0.912) values stems from the sensitivity of these indices to sample size (Marsh et al., 2004). In contrast, the strong fit of non-normed indices such as the CFI (0.943) and TLI (0.932) supports the model’s overall validity. Furthermore, the model’s modification indices (MI) and standardized error covariances were examined; only theoretically meaningful suggestions that could contribute to reducing measurement error were considered, and no error covariances without a theoretical basis were added. This approach ensured that the model maintained its statistical and theoretical integrity and was reported per confirmatory analysis standards (Table 3).

**Table 3:** Model fitting indicators.

	X^2^	df	*χ*^2^/df	RMSEA	CFI	GFI	NFI	TLI	IFI
Model	343.256	129	2.661	0.059	0.943	0.929	0.912	0.932	0.943

RMSEA, root mean square error of approximation; CFI, comparative fit index; GFI, goodness-of-fit index; NFI, normed fit index; TLI, Tucker-Lewis index. IFI, increased fit index.

Table 4 presents the results of the regression and mediation analyses examining the relationships between self-efficacy, psychological resilience, and life satisfaction.

**Table 4:** Results of regression and indirect effect analysis of the relationships between psychological resilience, self-efficacy and life satisfaction.

Psychological resilience (PR)						
Independent variables	*β*	SE	*t*	*p*	LLCI	ULCI
Constant	0.000	0.041	0.000	1.000	−0.0815	0.0815
Self-efficacy (SE)	0.517	0.051	10.040	0.000***	0.416	0.619
Model 1	R	R^2^	F	df1	df2	*p*
	0.417	0.173	100.816	1	479	0.000***

Life satisfaction (LS)						
Independent variables	*β*	SE	*t*	*p*	LLCI	ULCI
Constant	0.000	0.042	0.000	1.000	−0.0841	0.0841
Psychological resilience (PR)	0.141	0.041	3.373	0.008***	0.059	0.223
Self-efficacy (SE) (Direct Effect)	0.278	0.052	5.343	0.003**	0.175	0.380
Model 2	R	R^2^	F	df1	df2	*p*
	0.350	0.122	32.266	2	478	0.000***

Life satisfaction (LS)						
Independent variables	*β*	SE	*t*	*p*	LLCI	ULCI
Constant	0.000	0.043	0.000	1.000	−0.0850	0.0850
Self-efficacy (SE) (Total Effect)	0.351	0.047	7.347	0.000***	0.257	0.445
Model 3	R	R^2^	F	df1	df2	*p*
	0.318	0.101	53.985	1	479	0.000***
Indirect effect			*β*	SE	LLCI	ULCI
			0.073	0.025	0.024	0.126

**p < 0.01; ***p < 0.001.

In the first model, the effect of self-efficacy on resilience was tested, and a significant, positive relationship was found (*β* = 0.517, SE = 0.051, t = 10.040, *p <* 0.001). This model explained 17.3% of the variance in resilience (R^2^ = 0.173).

The second model examined the simultaneous effects of self-efficacy and psychological resilience on life satisfaction. Findings showed that both variables significantly and positively predicted life satisfaction (*β* - SE = 0.278, *β* - PR = 0.141; *p <* 0.01). The model explained 12.2% of the variance in life satisfaction (R^2^ = 0.122).

The third model tested the total effect of self-efficacy on life satisfaction. Self-efficacy significantly predicted life satisfaction (*β* = 0.351, SE = 0.047, t = 7.347, *p <* 0.001) and explained 10.1% of the variance (R^2^ = 0.101).

Finally, the mediation analysis determined that resilience partially mediated the effect of self-efficacy on life satisfaction (*β* = 0.073, SE = 0.025, 95% CI [0.024, 0.126]). According to Cohen’s (1988) benchmarks, the magnitude of this indirect effect corresponds to a small-to-moderate effect size, suggesting that while psychological resilience plays a meaningful mediating role, its practical impact is modest within the overall model. The fact that the confidence interval did not include zero indicates that the indirect effect was significant. Since the direct effect of self-efficacy on life satisfaction remained statistically significant even after including resilience as a mediator, the mediation was classified as partial. This means that resilience explains part of the effect of self-efficacy on life satisfaction, while a significant portion of the relationship remains direct. Overall, it was observed that self-efficacy contributed to life satisfaction both directly and indirectly through resilience. To complement the R^2^ interpretation, Cohen’s f^2^ effect sizes were calculated to assess each predictor’s contribution to the model. The f^2^ values indicated small effect sizes for both self-efficacy (f^2^ = 0.024) and psychological resilience (f^2^ = 0.024) on life satisfaction, according to Cohen’s (1988) guidelines. These results suggest that while both variables make statistically significant contributions to life satisfaction, their practical impact is modest, which is common in psychological models involving complex mediating mechanisms (Prentice and Miller, 1992). The explanatory power of the model was moderate in social science research (R^2^ = 0.17, 0.12, 0.10), and these rates were within the “practically significant” levels recommended by Cohen (2013) and Hair et al. (2009). LLCI–ULCI confidence intervals for the direct paths were also reported.

As seen in the model, self-efficacy affects life satisfaction directly and indirectly through psychological resilience. Self-efficacy was found to significantly and positively predict psychological resilience, while psychological resilience, in turn, increased life satisfaction. Indirect effect analyses indicate that psychological resilience partially mediates the relationship between self-efficacy and life satisfaction.

## Discussion

This study, which examines the mediating effect of psychological resilience on the relationship between self-efficacy and life satisfaction in student athletes, provides important insights into how psychological resources are associated with individuals’ life satisfaction. The model indicates that self-efficacy relates to life satisfaction both directly and indirectly through psychological resilience, with resilience serving a partial mediating function. These results highlight that self-efficacy and resilience are psychological mechanisms linked to life satisfaction among university student athletes and that the proposed model fits well with theoretical expectations. Nevertheless, several methodological limitations should be considered. The single-site sample, reliance on self-reported measures, and cross-sectional design prevent drawing conclusions about the directionality of relationships (Podsakoff et al., 2003). Moreover, the exclusion of variables such as gender, sport type, and participation level may limit the generalizability of the results. Although the study is grounded in Bandura’s self-efficacy theory, future research could examine alternative frameworks, including psychological capital and self-determination theory. Additionally, variables such as athletes’ competitive level, sport discipline, and training environment may moderate the relationships examined, which warrants further exploration.

The findings indicate that self-efficacy is positively associated with psychological resilience, consistent with Bandura’s (1997) theoretical assumptions. Individuals who perceive themselves as competent tend to approach difficulties with greater perseverance and adaptability. Self-efficacy appears to support motivation under stress, encourage viewing challenges as opportunities for growth, and promote resilient coping (Chrétien et al., 2024). Thus, self-efficacy and resilience emerge as complementary mechanisms that jointly support individuals’ psychological adjustment processes.

When viewed through broader theoretical perspectives, these findings align with both Bandura’s social cognitive theory and Self-Determination Theory (Deci and Ryan, 2013). The satisfaction of autonomy, competence, and relatedness needs is recognized as a key predictor of life satisfaction, and self-efficacy reflects how individuals fulfill their competence needs. Likewise, the Psychological Capital framework (Luthans et al., 2006) conceptualizes self-efficacy and resilience as essential positive psychological resources associated with greater wellbeing. Within these frameworks, the observed relationships between self-efficacy, resilience, and life satisfaction reinforce the view that these variables jointly explain individual differences in wellbeing.

Evidence from sports psychology supports the connection between self-efficacy and resilience. For instance, Zhang and Li (2025) found that a supportive sports environment is associated with higher resilience among athletes by enhancing their self-efficacy. Similarly, athletes with greater resilience report improved performance outcomes (Sehrawat et al., 2024), and strength-based therapeutic interventions have been shown to enhance both self-efficacy and life satisfaction (Parsakia et al., 2024). Although these cross-sectional findings cannot establish causality, they consistently reveal positive associations among these variables.

Recent studies have further examined these relationships in integrative models. For example, Zhong and Xu (2024) reported that the direct impact of physical exercise on life satisfaction is limited, but self-efficacy and resilience serve as significant mediators. Xu et al. (2021) similarly found that regular physical activity is linked to adaptive capacity, while Chang et al. (2023) observed that resilience predicts life satisfaction by buffering against stress and anxiety. Within the social cognitive framework, physical exercise is associated with stronger self-efficacy beliefs (Sabouripour et al., 2021), which in turn support the development of resilience (Tikac et al., 2021). This sequential pattern—self-efficacy promoting resilience and resilience relating to life satisfaction—has been replicated in multiple samples (Davis et al., 2011; Yu et al., 2025). Overall, these findings suggest that athletic participation may foster psychological resources that contribute to wellbeing, though other psychosocial factors such as social support, sense of belonging, and group cohesion also play important roles (Fletcher and Sarkar, 2016).

The present study also found that self-efficacy is positively related to life satisfaction both directly and indirectly through resilience. This pattern aligns with previous research showing that self-efficacy predicts life satisfaction in university students (Çakar, 2012; Ansari and Khan, 2015). University students with higher self-efficacy perceive themselves as more capable in academic and social contexts, which is associated with greater overall satisfaction with life. Sabouripour et al. (2021) similarly observed that resilience partially explains this association, a finding echoed by Yu et al. (2025). Zhong and Xu (2024) also demonstrated that the influence of physical activity on life satisfaction operates indirectly through self-efficacy and resilience. Moreover, Yu et al. (2025) emphasized that the strength of this relationship depends on exercise intensity, being more evident among individuals engaging in higher levels of activity. Regular physical exercise, therefore, appears to be related to enhanced self-efficacy, which in turn supports resilience and life satisfaction (Xu et al., 2021). These results, consistent with Bandura’s (1993, 1997) social cognitive theory, suggest that self-belief processes are associated with individuals’ perceived quality of life.

Prior studies also support the link between resilience and life satisfaction. For instance, Durak (2021) and Zhang et al. (2022) reported that individuals with higher resilience experience greater life satisfaction, while Gong et al. (2023) highlighted the role of resilience in mitigating academic stress and uncertainty. Wang and Liu (2022) found that resilience is related to improvements in quality of life by alleviating anxiety and depressive symptoms. From an athletic perspective, participation in sport and the activation of psychological mechanisms such as self-efficacy and resilience represent a meaningful process associated with life satisfaction. However, longitudinal and experimental designs are needed to clarify the direction and strength of these associations. Moreover, cultural context may influence these dynamics, as levels of individualism and collectivism can affect the expression and outcomes of self-efficacy (Schunk and DiBenedetto, 2020; Masten, 2018).

The mediation analysis indicated that resilience partially mediates the relationship between self-efficacy and life satisfaction. This suggests that self-efficacy relates to life satisfaction both directly and indirectly through resilience, while additional factors—such as social support, self-compassion, or sense of meaning—may also play roles in this process. Consistent with Zhang et al. (2022), self-efficacy appears positively associated with resilience, which in turn is linked to higher life satisfaction. Sabouripour et al. (2021) similarly found that resilience enhances the positive effect of self-efficacy on life satisfaction. Although the mediation is partial, the findings underscore the multifaceted nature of life satisfaction and the complementary roles of self-efficacy and resilience. Together, these psychological resources appear to sustain wellbeing among university student athletes. Designing athletic programs that simultaneously promote physical performance, self-efficacy, and resilience could therefore contribute to more comprehensive development and satisfaction. Future studies might incorporate additional individual (e.g., personality, motivation) and contextual (e.g., coaching support, social environment) variables to clarify the mechanisms underlying this partial mediation (Fletcher and Sarkar, 2016; Masten, 2018).

The established regression model explained 17.3% of the variance in life satisfaction, representing a moderate effect size in the social sciences (Durak, 2021). However, the remaining unexplained variance suggests that numerous psychological and social factors influence life satisfaction. Future studies would benefit from integrating variables such as self-compassion, social support, emotional intelligence, and a sense of meaning (Neff, 2011) while employing longitudinal or experimental designs to better understand the temporal and directional nature of these associations.

### Limitations and future research

This study has several limitations. First, data were collected via self-report measures, which could introduce a risk of social desirability bias or subjective bias in participants’ responses. Furthermore, collecting data from the same source and at the same time introduces the possibility of standard method variance. Second, because the study employed a cross-sectional design, the relationships between variables can be interpreted as causality. Third, low AVE values were found for some constructs in the measurement model, suggesting that the measurement validity of these constructs may be limited. Additionally, the scales used in the study had different Likert scales, which may have partially reduced comparability across measures. Another limitation of the study is the absence of control variables (e.g., gender, type of sport, training frequency) in the model. This raises the possibility that individual or contextual differences may have influenced the results. The exclusion of control variables such as age, gender, and years of sport participation may have limited the internal validity of the structural model by not accounting for individual differences. Future studies are encouraged to include these control variables as covariates or moderators to better understand their potential influence on psychological resilience, self-efficacy, and life satisfaction. Furthermore, the fact that the sample consisted solely of university student athletes limits the generalizability of the findings. Furthermore, the study was conducted within a specific cultural context; therefore, the relationships between psychological resilience, self-efficacy, and life satisfaction may manifest differently across cultures. Therefore, considering cultural and contextual factors would significantly contribute to future studies. Future research using longitudinal designs could primarily examine the direction and change in the relationships between self-efficacy, psychological resilience, and life satisfaction over time. Furthermore, adopting mixed-method approaches and obtaining qualitative findings alongside quantitative data could contribute to a deeper understanding of the impact of these variables on individual experiences. Future studies could include psychological variables such as social support, self-esteem, and optimism, and test their mediating or moderating roles. For example, the moderated mediating effect of social support on the relationship between psychological resilience and life satisfaction could be investigated. Additionally, the effects of practices that improve self-efficacy and psychological resilience on life satisfaction should be tested through experimental or intervention-based research. Comparative studies conducted with different age groups, professional athletes, or diverse cultural samples will increase the generalizability of the findings. Furthermore, the findings of this study also offer important practical implications for educators and coaches. Educators should create learning environments that support students’ self-efficacy levels, make success experiences visible, and reinforce psychological resilience through positive feedback and modeling techniques. Such approaches can enhance students’ resilience to academic stress and support their life satisfaction. For coaches, practices such as goal setting, emotional support, strengthening team communication, and developing feedback mechanisms that foster self-efficacy are prominent practices for enhancing psychological resilience. Coaches who acknowledge athletes’ efforts, foster a trusting communication climate, and foster a supportive training culture that recognizes the right to make mistakes can strengthen athletes’ self-efficacy and life satisfaction. Therefore, developing psychoeducational programs aimed at improving self-efficacy and psychological resilience is recommended for educational institutions and sports organizations. Such practices will provide a framework that supports individual performance, psychological wellbeing, and sustainable life satisfaction.

## Data Availability

The raw data supporting the conclusions of this article will be made available by the authors, without undue reservation.
